# Single-cell ATAC-Seq in human pancreatic islets and deep learning upscaling of rare cells reveals cell-specific type 2 diabetes regulatory signatures

**DOI:** 10.1016/j.molmet.2019.12.006

**Published:** 2019-12-20

**Authors:** Vivek Rai, Daniel X. Quang, Michael R. Erdos, Darren A. Cusanovich, Riza M. Daza, Narisu Narisu, Luli S. Zou, John P. Didion, Yuanfang Guan, Jay Shendure, Stephen C.J. Parker, Francis S. Collins

**Affiliations:** 1 Department of Computational Medicine & Bioinformatics, University of Michigan, Ann Arbor, MI, 48109, USA; 2 National Human Genome Research Institute, National Institutes of Health, Bethesda, MD, 20892, USA; 3 Department of Genome Sciences, University of Washington, Seattle, WA, 98109, USA; 4 Department of Human Genetics, University of Michigan, Ann Arbor, MI, 48109, USA

**Keywords:** Islet, Epigenomics, Deep learning, Single cell, Chromatin, Type 2 diabetes, ATAC-seq, assay for transposase accessible chromatin sequencing, GWAS, genome-wide association study, eQTL, expression quantitative trait loci, GCG, glucagon, INS, insulin, SST, somatostatin

## Abstract

**Objective:**

Type 2 diabetes (T2D) is a complex disease characterized by pancreatic islet dysfunction, insulin resistance, and disruption of blood glucose levels. Genome-wide association studies (GWAS) have identified > 400 independent signals that encode genetic predisposition. More than 90% of associated single-nucleotide polymorphisms (SNPs) localize to non-coding regions and are enriched in chromatin-defined islet enhancer elements, indicating a strong transcriptional regulatory component to disease susceptibility. Pancreatic islets are a mixture of cell types that express distinct hormonal programs, so each cell type may contribute differentially to the underlying regulatory processes that modulate T2D-associated transcriptional circuits. Existing chromatin profiling methods such as ATAC-seq and DNase-seq, applied to islets in bulk, produce aggregate profiles that mask important cellular and regulatory heterogeneity.

**Methods:**

We present genome-wide single-cell chromatin accessibility profiles in >1,600 cells derived from a human pancreatic islet sample using single-cell combinatorial indexing ATAC-seq (sci-ATAC-seq). We also developed a deep learning model based on U-Net architecture to accurately predict open chromatin peak calls in rare cell populations.

**Results:**

We show that sci-ATAC-seq profiles allow us to deconvolve alpha, beta, and delta cell populations and identify cell-type-specific regulatory signatures underlying T2D. Particularly, T2D GWAS SNPs are significantly enriched in beta cell-specific and across cell-type shared islet open chromatin, but not in alpha or delta cell-specific open chromatin. We also demonstrate, using less abundant delta cells, that deep learning models can improve signal recovery and feature reconstruction of rarer cell populations. Finally, we use co-accessibility measures to nominate the cell-specific target genes at 104 non-coding T2D GWAS signals.

**Conclusions:**

Collectively, we identify the islet cell type of action across genetic signals of T2D predisposition and provide higher-resolution mechanistic insights into genetically encoded risk pathways.

## Highlights

• Single cell chromatin accessibility profiles obtained for 1,456 human pancreatic islet cells.
• Unique α, β, and δ cell chromatin accessibility signatures identified.
• Deep learning approach accurately predicts chromatin accessibility in rare cells.
• Nominate effector cell types, causal variants and target genes at diabetes GWAS loci.

## 1 Introduction

Pancreatic islets consist of a cluster of at least five different endocrine cell types (alpha, beta, delta, gamma, and epsilon), each producing a unique hormone in a distinct but coordinated manner [1]. Collectively, these clusters of cells work together to maintain insulin production and glucose homeostasis. Disruption of the complex interplay between the cell types, their organization, and their underlying regulatory interaction is known to be associated with type-2-diabetes (T2D) pathophysiology [2]. However, the exact cellular mechanisms through which different risk factors contribute to disease risk are not completely understood. Using GWAS and eQTL mapping approaches, recent studies have discovered >400 independent signals (>240 loci) associated with T2D and T2D-associated traits [3], although remarkably, more than 90% localize to non-protein-coding regions of the genome [4]. Growing evidence suggests that many of these variants likely influence the RNA expression and cellular function of human pancreatic islets by altering transcription factor binding, critical components of a cellular regulatory network [[5], [6], [7], [8], [9]].

High-throughput epigenomic profiling methods such as ATAC-seq [10] and DNase-seq [11] have enabled profiling of chromatin accessibility across samples in a tissue-wide manner, providing the opportunity to identify millions of context-specific regulatory elements. However, these bulk measurements of chromatin accessibility limit the precise understanding of how tissue heterogeneity and multiple cell types in the population contribute to overall disease etiology [12]. Recent advances in single-cell transcriptomic and epigenomic profiling methods have enabled an unbiased identification of cell-type populations and regulatory elements in a heterogeneous biological sample. By mapping the chromatin-regulatory landscape at a single-cell resolution, recent single-nuclei studies have demonstrated the potential to discover complex cell populations, link regulatory elements to their target genes, and map regulatory dynamics during complex cellular differentiation processes [[13], [14], [15], [16]]. The pancreatic islet gene expression landscape has been investigated at single-cell resolution in existing studies [17,18], but chromatin accessibility studies have been limited to fluorescence-activated cell sorting (FACS) methods to obtain cell-type populations [19,20]. FACS-based methods will miss identification of unknown or rarer cell-populations and are unable to produce pure cell-type populations due to reliance on the specificity of cell surface markers [21,22].

In this study, we present a genome-wide map of chromatin accessibility in >1,600 nuclei derived from a human pancreatic islet sample using single-cell combinatorial indexing ATAC-seq (sci-ATAC-seq) [23]. Sci-ATAC-seq enables us to deconvolve cell populations and identify cell-type-specific regulatory signatures underlying T2D. Notably, T2D GWAS SNPs are significantly enriched in beta cell-specific and across cell-type shared islet open chromatin, but not in alpha or delta cell-specific open chromatin. We then develop a novel deep learning-based strategy using concepts borrowed from image upscaling methods to improve signal recovery and feature reconstruction for low abundance cell populations and apply it successfully to delta cells (<5% of the total islet population) identified in our study. We anticipate that our deep learning method will enable analysis of heterogeneous tissues that may be harder to obtain in large numbers or contain rare subpopulations. We validate our findings against multiple independent and orthogonal datasets that consistently support the high quality and reproducibility of our results. Collectively, these results identify the islet cell type of action across genetic signals of T2D predisposition and provide higher-resolution mechanistic insights into genetically encoded pathophysiology.

## 2 Materials and methods

### 2.1 Bulk islet ATAC-seq

#### 2.1.1 Sample processing

Human pancreatic islet samples were procured and processed as described by Varshney et al. [8]. Briefly, the islets were obtained from the National Disease Research Interchange (NDRI) and processed according to the NHGRI institutional review board-approved protocols. The islets were shipped overnight from the distribution center. Upon receipt, we pre-warmed the islets to 37° in shipping media for 1–2 h before harvest. A total of ∼50–100 islet equivalents (IEQs) were harvested and transposed in triplicate following the methods of Buenrostro et al. [10]. The ATAC-seq library was barcoded and sequenced 2 × 125 bp on a HiSeq 2000.

#### 2.1.2 ATAC-seq analysis

Sequencing adapters were trimmed using CTA (v0.1.2) [24] and aligned to the hg19 reference genome using BWA-MEM (v0.7.15, r1140, options: -I 200, 200, 5000) [25]. Picard MarkDuplicates (v2.18.27) was used for duplicate removal and Samtools [26] was used to filter for autosomal, properly paired, and mapped read pairs with mapping quality ≥ 30 (v1.9, options: -f3 -F3340 -q30). Replicates across each sample were merged into a single file using Samtools merge. For peak calling, each sample was downsampled to 25 million (M) reads and converted to a BED file. We then used MACS2 [27] to call broad peaks (v2.1.1.20160309; options: --nomodel --broad --shift -100 --extsize 200 --keep-dup all --SPMR) and removed those with FDR > 0.05 and overlapping with ENCODE hg19 blacklists [28]. ATAC-seq coverage tracks were displayed using the UCSC Genome Browser and Integrative Genomics Viewer (IGV). Summary statistics were calculated using ataqv (v1.0) [29] and are available in interactive and downloadable formats online (Table S7). For comparative purposes, we performed the same read trimming, alignment, filtering, downsampling, and peak calling steps on publicly available ATAC-seq data (Table S1). Peaks from each sample were merged to create a master peak set and Spearman's correlation was computed on the RPKM-normalized read-count matrix.

#### 2.1.3 Determination of high-confidence peaks

We randomly sampled 2.5 M reads from each sample using Samtools view and pooled them into one file so that each sample was equally represented. Peaks were called on the pooled file as discussed in the previous paragraph. We then determined the number of samples overlapping with each master peak using peaks called on individual samples.

#### 2.1.4 Overlap of reads with ChromHMM states

We tested for enrichment of the ATAC-seq peaks across 13 islet-specific chromatin states using Genomic Association Tester (GAT) [30]. We ran GAT (v1.3.5, options: --number-samples 10,000) and filtered the chromatin states with no significant enrichment (Bonferroni adjusted p-value < 0.05) of their peaks. Fold enrichment (log_2_) values across the chromatin states were clustered using hierarchical clustering of the correlation matrix.

### 2.2 Sci-ATAC-seq analysis

#### 2.2.1 Sample processing

We used the combinatorial cellular indexing method to generate single-nuclei chromatin accessibility data as previously described by Cusanovich et al. [23]. Briefly, a suspension of islet cells was obtained and pelleted for 5 min at 4 °C 500×*g*. The medium was aspirated and the cells were washed once in 1 ml PBS. The cells were pelleted again for 5 min at 4 °C 500×*g* and then resuspended in 1 ml of cold lysis buffer (10 mM Tris–HCl, pH 7.4, 10 mM NaCl, 3 mM MgCl_2_, and 0.1% IGEPAL CA-630 supplemented with 1× protease inhibitors (Sigma P8340)). Nuclei were maintained on ice whenever possible after this point. Then 10 μl of 300 μM DAPI stain was added to 1 ml of lysed nuclei for sorting. To prepare for sorting, 19 μl of freezing buffer (50 mM Tris at pH 8.0, 25% glycerol, 5 mM MgOAc_2_, 0.1 mM EDTA supplemented with 5 mM DTT, and 1× protease inhibitors (Sigma P8340)) was aliquoted into each well of a 96-well LoBind plate. A total of 2,500 DAPI^+^ nuclei (single-cell sensitivity) were sorted into each well of the plate containing freezing buffer. The plate was sealed with a foil plate sealer and then snap frozen in liquid nitrogen. The frozen plate was then transferred directly to a −80 °C freezer. The sample was subsequently shipped from NIH to UW overnight on dry ice. The plate was then thawed on ice and supplemented with 19 μl of Illumina TD buffer and 1 μl of custom-indexed Tn5 (each well received a different Tn5 barcode). The nuclei were tagmented by incubating at 55 °C for 30 min. The reaction was then quenched in 20 mM EDTA and 1 mM spermidine for 15 min at 37 °C. The nuclei were then pooled and stained with DAPI again. A total of 25 DAPI^+^ nuclei were then sorted into each well of a 96-well LoBind plate containing 11.5 μl of Qiagen EB buffer, 800 of μg/μl BSA, and 0.04% SDS. Then 2.5 μl of 10 μM P7 primers were added to each sample and the plate was incubated at 55 °C for 15 min. Then 7.5 μl of NPM was added to each well. Finally, 2.5 μl of 10 μM P5 primers were added to each well and the samples were PCR amplified at following cycles: 72 °C for 3 min, 98 °C for 30 s, then 20 cycles of 98 °C for 10 s, 63 °C for 30 s, and 72 °C for 1 min. The exact number of cycles was determined by first doing a test run on 8 samples on a real-time cycler with SYBR Green (0.5 × final concentration). The PCR products were then pooled and cleaned on Zymo Clean & Concentrator 5 columns (the plate was split across 4 columns), eluted in 25 μl of Qiagen EB buffer, and then all 4 fractions were combined and cleaned using a 1× AMpure bead cleanup before eluting in 25 μl of Qiagen EB buffer again. The molar concentration of the library was then quantified on a Bioanalyzer 7500 chip (including only fragments in the 200–1000 bp range) and sequenced on an Illumina NextSeq at 1.5 pM concentration.

#### 2.2.2 QC and pre-processing

*Step 1. Barcode correction and filtering*. Each barcode consisted of four 8-bp-long indexes (i5, i7, p5, and p7). Reads with barcode combinations containing more than 3 edit distances for any index were removed. If a barcode was within 3 edits of an expected barcode and the next best matching barcode was at least 2 edits further away, we corrected the barcode to its best match. Otherwise, the barcode was classified as ambiguous or unknown.

*Step 2. Adapter trimming and alignment*. Adapters were removed using Trimmomatic [31] with NexteraPE adapters as input (ILLUMINACLIP:NexteraPE.fa:2:30:10:1:true TRAILING:3 SLIDINGWINDOW:4:10 MINLEN:20) and aligned to an hg19 reference using BWA-MEM (v0.7.15, r1140, options: -I 200, 200, 5000) [25]. The final alignment was filtered using Samtools to remove unmapped reads and reads mapping with quality <10 (-f3 -F3340 -q10) as well as reads that were associated with ambiguous or unknown barcodes.

*Step 3. Deduplication and nuclei detection*. Duplicates from the pruned file were removed using a custom Python script on a per-nucleus basis. Using the distribution of reads per barcode, we applied bi-clustering as implemented in the mclust [32] R package to differentiate between the background barcodes and barcodes that corresponded to a nucleus. Using a list of non-background barcodes, we split the aggregate BAM file into constituent BAM files corresponding to each barcode representing a single nucleus using a custom Python script.

*Step 4. Quality assessment of each single nucleus*. For each single nucleus, we computed ATAC-seq quality metrics such as the fragment length distribution, transcription start site (TSS) enrichment, short-to-mononucleosomal reads ratio, total autosomal reads, and fraction of reads overlapping peaks. We removed nuclei with a) total reads outside the 5%–95% range (34578–226755) of all of the nuclei and b) TSS enrichment of <2.7 (5% tile) from further downstream analysis.

*Step 5. Aggregate sci-ATAC-seq peaks*. We pooled reads from the filtered barcodes from the previous steps to create an aggregate BAM file. Peaks were called and filtered as described previously in the Bulk islet ATAC-seq analysis section.

### 2.3 Cluster analysis

#### 2.3.1 Feature selection and clustering

We generated a list of TSS distal peaks (>5 kb away from the nearest TSS based on RefSeq genes [33]) from the aggregate sci-ATAC-seq data. For each nucleus, we counted the number of reads overlapping the peaks using the Rsubread package [34]. We then adopted a logistic regression approach to remove peaks in which the binarized accessibility across nuclei was significantly associated (Bonferroni corrected p-value < 0.05) with the sequencing depth. This approach should help reduce the bias associated with the sequencing depth, as the remaining peaks are no longer associated with this technical factor, a strategy that has been successfully implemented in single-cell RNA-seq data analysis [35]. The resulting count matrix was RPKM-normalized and reweighted using the term-frequency and inverse document frequency (TF-IDF) method [13]. To accomplish this, we first weighted all the sites for individual nuclei by the total number of sites accessible in that cell (“term frequency”). We then multiplied these weighted values by log (1 + the inverse frequency of each site across all cells), the “inverse document frequency.” The TF-IDF transformed matrix was then reduced to 30 principal components using principal component analysis (PCA) and used as input to generate two-dimensional embedding using the uniform manifold approximation method (UMAP, n_neighbors = 20) [36]. We identified clusters in the two-dimensional embedding in an unsupervised manner using a density-based clustering method (HDBSCAN, minPts = 20) as implemented in the DBSCAN R package [37].

#### 2.3.2 Cell identity assignment and validation

The cell identities were assigned based on de facto cell-type-specific hormone markers: *INS-IGF2* (beta), *GCG* (alpha), and *SST* (delta) among others. A marker gene was considered to be present in a nuclei if a read mapped within 5 kb of the GENCODE (v19) gene body annotation [38]. For additional verification of the cell identity, we computed the RPKM-normalized aggregate ATAC-seq signal across cell-type marker genes reported in two independent islet scRNA-seq studies [17,39]. Finally, we evaluated the enrichment of the cells from each cell-type cluster relative to their expected population proportion using a two-sided binomial test across 10 bins of sequencing depth (∼145 cells/bin).

### 2.4 Deep learning signal and peak upscaling

#### 2.4.1 Model design, training, and validation strategy

The U-Net model [40] takes input sequences and outputs prediction sequences. The goal of model training is to reduce the error between the prediction output and a representation of the ground truth. For signal upscaling, the input sequence was base-wise scores of BAM pileups (read-depth) corresponding to a subsample of n cells (randomly sampled from 600 cells) and the output sequence was base-wise scores of BAM pileups using reads from all 600 cells. Peak upscaling not only uses the subsampled BAM pileup scores as inputs but also uses the binary base-wise values from calling peaks with MACS2 on the subsampled BAM alignments. The output sequences for peak upscaling are the binary base-wise values from calling peaks with MACS2 on the data. We created two models, each separately based on the data from the alpha and beta cells. Because both had different numbers of constituent single nuclei, we matched the size of the output dataset by randomly sampling 600 cells from each cell-type cluster. The input datasets were created by sampling n cells from the set of 600 cells such that the total number of reads was similar across both models. We did not set any explicit constraints on the number of peaks to be called by this approach.

The network architecture of the U-Net model used in this study is illustrated in Fig. S2A. It consisted of a contracting convolutional path (left side) and an expansive deconvolutional path (right side). The contracting path consisted of repeated applications of two kernel size 11 convolutions (unpadded convolutions) with rectified linear unit (ReLU) activation and a kernel size 2 max pooling operation with stride 2 for downsampling. Each downsampling step halves the length of the activation sequence while doubling the number of feature channels. Every step in the expansive path consists of a kernel size 2 deconvolution layer with a linear activation function that halves the number of feature channels, a concatenation with the correspondingly cropped feature map from the contracting path, and two kernel size 11 convolution layers with ReLU activations. The cropping is necessary due to the loss of border sequence steps in a non-padded convolution. At the final layer, a kernel size 1 convolution with either an ReLU (for signal upscaling) or sigmoid (for peak upscaling) activation function generates the sequence of predictions. Due to the use of unpadded convolutions, the prediction sequence is shorter than the input sequence by a constant border width. Although the U-Net model can accept arbitrary length input sequences, we fixed all of the training samples to have a length of 6700, which resulted in output prediction sequences with a length of 4820. In total, the network had five steps each in the contracting and expansive paths for a total of 27 convolutional layers and 8,998,529 training parameters. The model was implemented using Keras [41] with the TensorFlow [42] backend, and the experiments were run using Titan Xp and GTX 1080 Ti GPUs.

To reduce overfitting, we split chromosomes into training, validation, and testing sets. The model was fit using the ADAM optimizer [43] with a learning rate of 1e-5 and a batch size of 128 for 50 epochs. Separate loss functions, and hence models, were used to solve signal and peak upscaling. For signal upscaling, we used the mean squared error base-wise loss function. For peak upscaling, the loss function was the sum of the cross-entropy base-wise loss and the Dice coefficient loss, also known as the F1 score. We used the mean average precision, a common evaluator for object detection, and Pearson's correlation as the output evaluation metrics for the peak and signal upscaling, respectively. The downscaling and model training were repeated for n = 5, 10, 28, 50, 100, 200, 300, 400, and 500 cells.

#### 2.4.2 Generating upscaled peaks

To select a subset of high-confidence peaks from the predicted model output, we adopted a post-hoc approach in which we compared the number of cell-type-specific peaks for alpha, beta, and delta cells, and chose a threshold in which they had a similar number. For the predicted delta cell peaks, we combined the results from the alpha and beta models at the same threshold using bedtools [44] intersect (v.2.27.1) after filtering for the chosen threshold.

### 2.5 Cell-type-specific peaks analysis

#### 2.5.1 Cell-type-specific peaks

Peaks specific to each cell type were obtained by comparing the peaks in one cell-type with all of the other cell types using bedtools.

#### 2.5.2 T2D GWAS SNPs enrichment

Enrichment of T2D-associated GWAS SNPs from DIAMANTE [3] was tested using GREGOR (v1.3.1) [45]. Specifically, we used the following parameters: r2 threshold (for inclusion of SNPs in LD with the diabetes-associated GWAS SNPs) = 0.80, LD window size = 1 Mb, and minimum neighbor number = 500. The p-values were adjusted according to the Bonferroni threshold for multiple testing burdens.

#### 2.5.3 Conditional fGWAS enrichment analysis

We used fGWAS [46] to model the shared properties of loci affecting a trait. We ran fGWAS (v0.3.6) with DIAMANTE T2D GWAS summary data and cell-type ATAC-seq peaks from three cell types as input annotations. For each individual annotation, the output model provided maximum likelihood enrichment parameters and annotations were considered significantly enriched if the parameter estimates and 95% confidence interval (CI) did not overlap zero. We then used fGWAS to run a conditional analysis in a pair-wise manner in which the enrichment of one model was conditionally evaluated on the output models from the other annotations.

#### 2.5.4 Validating cell-type peaks using scRNA-seq signature genes

We evaluated the enrichment of scRNA-seq derived signature genes (scRSGs) in 28-cell MACS2 and upscaled peak calls using GAT [30]. We ran GAT (v1.3.5, options: --number-samples 10,000) with the union of all peaks as workspaces and scRSGs as segments.

#### 2.5.5 Transcription factor motif enrichment

We used motif PWN scans from [47]. Briefly, we used biallelic SNPs and short indels from the 1,000 Genomes Project (release v5) [48] to generate comprehensive scans with FIMO [49] using the background nucleotide frequencies from hg19 and a p-value < 1e-4. We only kept motif instances that intersected mappable regions and did not intersect blacklisted regions. We then tested for the enrichment of motifs across cell-type-specific peaks using GAT (v1.3.5, options: --number-samples 100,000) [30]. We used a union of the top 100 motifs (by log fold enrichment) for each annotation and clustered them using hierarchical clustering.

### 2.6 Linking SNPs to target genes

#### 2.6.1 Cicero co-accessibility analysis

To link TSS distal ATAC-seq peaks with target genes, we used Cicero [50], which identifies co-accessible pairs of DNA elements using single-cell chromatin accessibility data. We used these results to infer connections between regulatory elements and their target genes. We ran Cicero (v1.0.15, default parameters) with cells from the alpha and beta cell clusters separately. To accomplish this, we first called peaks on each cluster and counted the number of reads per nuclei overlapping the peaks. The resulting count matrix was used as input to Cicero along with the UMAP projection for each cluster. Finally, to decide a threshold for filtering co-accessible peak pairs, we computed the Fisher odds ratio for the enrichment of co-accessible peaks vs distance-matched non-co-accessible peaks (co-accessibility < 0) with three different three-dimensional chromatin looping data sets: islet Hi-C [51], islet promoter capture Hi-C (pcHi-C) [52], and EndoC Pol2 ChIA-PET anchors [53]. For overlap, we checked whether both the ends of the Cicero loops intersected with both of the anchors from the experimental chromatin looping data. Public epigenome browser session links are included in Table S7.

#### 2.6.2 T2D GWAS SNP overlap analysis

To link T2D GWAS SNPs with target genes, we utilized 380 independent GWAS signals from DIAMANTE that were genetically fine-mapped to 99% credible sets using a Bayesian approach. In this framework, each SNP has a posterior probability for being causal for the association in that region. These posterior probabilities are the ratio of evidence for each variant vs all of the others, which makes it easy to directly compare the variants. A genetic credible set is then defined as the minimum set of SNPs that contains all SNPs with a probability greater than or equal to 0.01. We filtered the SNPs within each set to have >0.05 posterior probability of association (PPAg). We then checked for each GWAS signal whether SNPs passing the criteria mapped within 1 kb of cell-type-specific ATAC-seq peaks. To obtain Cicero target genes, we checked if an ATAC-seq peak was a) within 1 kb of a variant, b) outside the 1 kb range of a RefSeq TSS, and (c) linked to ATAC-seq peaks within 1 kb of a RefSeq TSS. The binary overlap matrix was clustered using hierarchical clustering with the binary distance method. Tables containing the number of SNPs within each credible set and specific variants overlapping cell-type-specific ATAC-seq peaks are available in Table S8 and Table S9.

## 3 Results

### 3.1 Sci-ATAC-seq captures tissue relevant characteristics similar to bulk ATAC-seq

**Figure 1:**
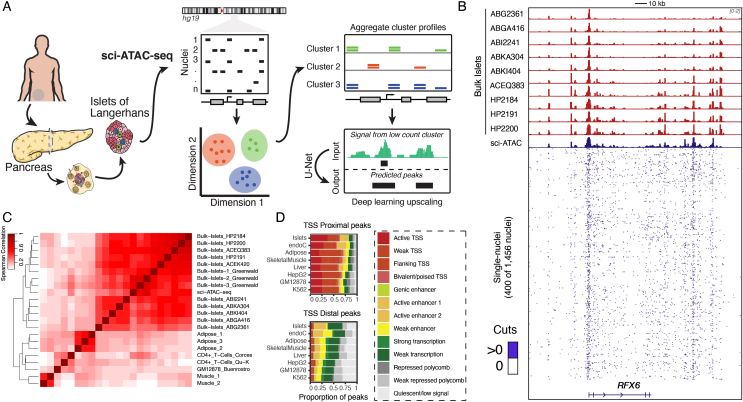
**Schematic of sci-ATAC-seq study.** (A) Sci-ATAC-seq protocol for generating single-nuclei ATAC-seq data from a pancreatic islet sample. The data are then used to identify constituent cell types and use a deep learning model to predict peaks on the clusters with fewer nuclei count. (B) ATAC-seq signal tracks for 10 bulk islet samples and the sci-ATAC-seq islet sample. Bottom tracks show the signal across a random subset of up to 400 single nuclei. Signal tracks are normalized to one million reads and scaled between 0 and 2. (C) Spearman's correlation between aggregate sci-ATAC-seq, 13 bulk islets, 3 adipose, 2 muscle, 2 CD4+ T-cells, and 1 GM12878 sample (see Table S1). (D) Distribution of aggregate sci-ATAC-seq TSS proximal and distal peaks across bulk islet derived ChromHMM segmentations.

Pancreatic islets represent approximately 1–2% (by mass) of total pancreatic tissue [1] and therefore require specialized approaches to isolate in a manner that maintains viability. We obtained a highly pure (>95% purity and >92% viability) sample of human pancreatic islet tissue from one individual (cadaveric donor, female, 43 years old, and non-diabetic) and profiled chromatin-accessibility using the sci-ATAC-seq protocol [23] as described previously (Figure 1A, Table S2). In total, we obtained 1,690 single-cell ATAC-seq datasets with depths ranging from 17,667 to 415,237 (median: 79,482) reads per nucleus and TSS enrichment from 0.77 to 9.80 (median: 3.91) after removing the background barcodes (Fig. S1A). For quality assessment of each single nucleus, we reasoned that the total reads and TSS enrichment values are more suitable metrics for identifying nuclei with poor signal-to-noise ratio than using fraction of reads in peaks as the latter may bias counts for under-represented cell-type populations in the analysis (Figs. S1B–C). Based on these criteria, we obtained high-quality sci-ATAC-seq data for 1,456 single nuclei. In addition to the sci-ATAC-seq data, we generated high-quality bulk ATAC-seq data for 10 islet samples with >47 M reads and >4.4 TSS enrichment per sample (Table S3). Using our approach to identify high-confidence (master) peak calls across the samples (see Methods), we obtained 106,460 bulk islet accessible chromatin peaks.

We then compared the aggregate islet sci-ATAC-seq data with bulk ATAC-seq samples from islets and other tissues. To accomplish this, we called 156,311 peaks on the aggregate sci-ATAC-seq. We found that the aggregate sci-ATAC-seq profiles were concordant and clustered together with the other bulk islet samples, indicating that aggregate sci-ATAC-seq can capture chromatin accessibility in a manner equivalent to bulk ATAC-seq assays (Figure 1B–C, Figure S1D–E). Further, to understand whether the aggregate sci-ATAC-seq peaks captured islet-specific regulatory features, we compared the distribution of peaks across ChromHMM chromatin state maps across eight different tissues, including islets and the EndoC human beta cell line [8]. We found that the islet sci-ATAC-seq peaks overlapped active TSS and active enhancer segmentations in the islet and EndoC (a beta cell line) chromatin state maps to a larger extent compared to other tissues (Figure 1D). Because ChromHMM enhancer states are driven by H3K27ac marks and are known to be associated with tissue-specific enhancer activity [5,54], our results indicate that the sci-ATAC-seq data captured the underlying islet-specific chromatin architecture similar to bulk islet ATAC-seq assays. Overall, these results indicate that our aggregate islet sci-ATAC-seq data are of high quality and suggests that the individual nuclei could reveal cell-specific patterns of the constituent cell types.

### 3.2 Sci-ATAC-seq reveals constituent cell types in pancreatic islets

**Figure 2:**
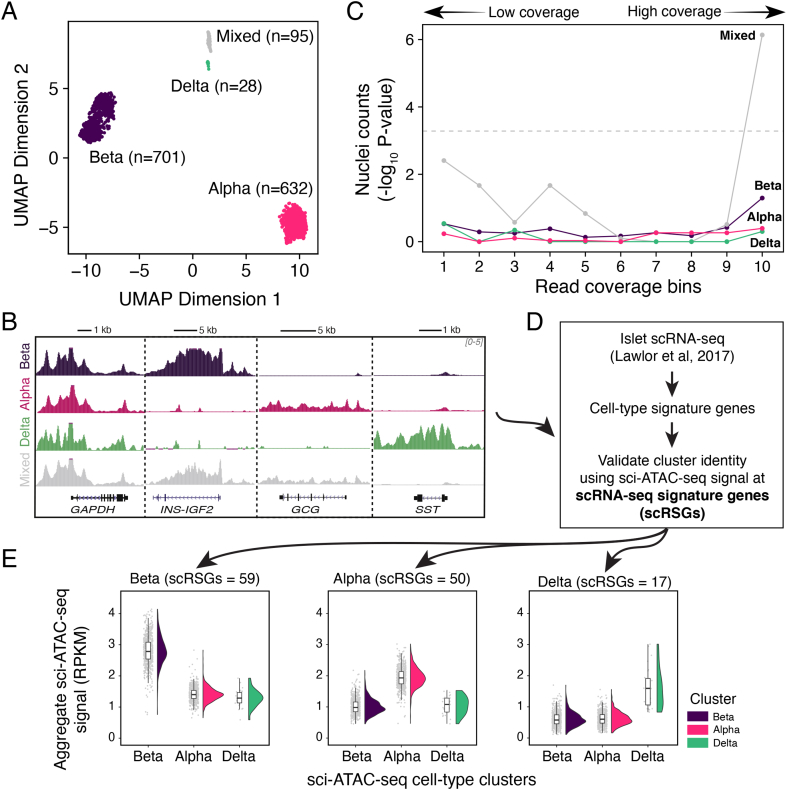
**Clustering and identification of cell-type clusters in sci-ATAC-seq data.** (A) UMAP projection with clustering of 1,456 single-nuclei islets represented by each single point into four clusters as identified by density-based clustering. (B) Enrichment of cells from each cluster relative to their expected population proportion across different read sequencing depth bins. Sequencing depth increases with the bin number. (C) Genome browser tracks showing signals at different cell-type marker loci: alpha (*GCG*), beta (*INS-IGF2*), delta (*SST*), and a housekeeping gene (*GAPDH*). Tracks are normalized to one million reads and scaled between 0 and 5. (D) Overview of the independent cluster verification scheme utilizing cell-type signature genes as identified by an islet scRNA-seq study by Lawlor et al. (2017). (E) Plot of aggregate ATAC-seq signals (RPKM) at scRNA-seq derived cell-type signature genes for alpha, beta, and delta cells. Number of signature genes for each cell type indicated in the title.

The aggregate sci-ATAC-seq profile of the islet is constituted of signals from distinct cell types. To identify these cell types, we leveraged the observation that TSS distal regions capture cell-type-specific accessibility patterns and are effective at classifying constituent cell types [55]. We adopted a multi-step process to robustly detect and identify islet subpopulations (see Methods, Table S4). This approach produced four distinct clusters (Figure 2A). To assign a cell-type identity to the clusters, we merged the nuclei in each cluster to create aggregate chromatin accessibility profiles and systematically examined the patterns of accessibility at multiple cell-type marker loci. We found three clusters that had distinct chromatin-accessibility patterns at *GCG*, *INS-IGF2*, and *SST* loci corresponding to three major islet cell types: alpha, beta, and delta cells (Figure 2B). The fourth cluster (95 nuclei, ∼7% of all nuclei) showed a “mixed” cell-type appearance as shown by signals at multiple cell-specific markers. We reasoned that these were likely to be nuclei doublets resulting from barcode collisions inherent to the combinatorial indexing protocol and thus should have skewed ATAC-seq read coverage. Indeed, we observed that the nuclei assigned to the mixed cell cluster were significantly (nominal p-value = 7.3e-7, binomial test) enriched in the high sequencing depth bin relative to the nuclei from other clusters (Figure 2C). As such, these nuclei were removed from further analyses, yielding a total of 1,361 nuclei with 51%, 47%, and 2% identified as beta, alpha, and delta, respectively. These estimates agree with the existing estimates of pancreatic islet cell-type proportions observed via confocal microscopy or single-cell transcriptomics experiments [17,39,56,57]. We additionally validated the identity of our clusters using findings from two independent islet scRNA-seq studies [17,39]. For both studies, we observed that our chromatin accessibility profiles across cell-type signature genes were enriched with our assigned cluster identities (Figure 2E, Fig. S1F).

We then analyzed the chromatin accessibility profile for each cell-type cluster. To accomplish this, we aggregated the nuclei within each cluster and identified 129,046 peaks for the alpha cells and 120,116 peaks for the beta cells using MACS2. However, because the delta cluster had only 28 cells (corresponding to ∼2 M reads), we reasoned that MACS2 would not perform ideally on data with such low depth. Indeed, we identified only 49,293 peaks using MACS2 on the delta cell aggregate reads.

### 3.3 Deep learning enables robust peak calls on less abundant delta cells

**Figure 3:**
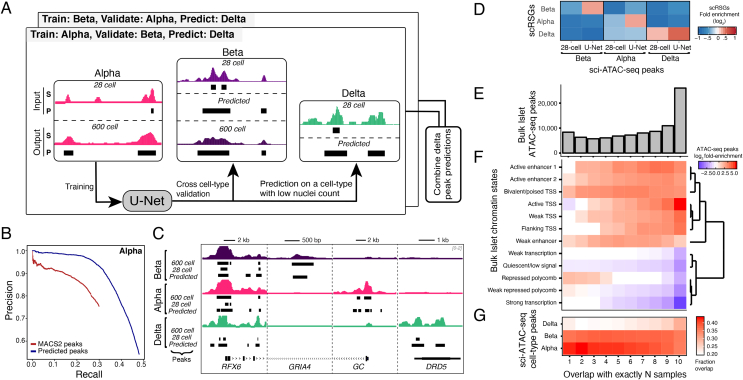
**Deep learning upscaling from sparse low-count nuclei clusters.** (A) Schematic of the U-Net training scheme. Two models are depicted in the illustration: one trained on alpha cells data as input and other trained on beta cells as input. Delta cell peak predictions from both models are combined to obtain final predictions (see Methods). (B) Precision-recall curve comparing peak calls from MACS2 on downscaled data (alpha cell type) with predicted peak calls from the 28-cell U-Net model (trained on beta, predicted on alpha). (C) Example loci illustrating peak upscaling with the model. For each cell type, four tracks are shown: full signal track, peak calls on full data, peak calls on subsampled data, and predicted peak calls. The predicted peak calls are obtained from a model trained on a different cell type. For delta predicted peak calls, intersection of prediction from both alpha and beta models are shown. Signal tracks normalized to one million reads and scaled between 0 and 2. (D) Fold enrichment (log_2_) of single-cell RNA-seq derived signature genes (scRSGs) in 28-cell MACS2 and U-Net predicted peaks for three cell types. (E) Reproducibility of master peaks from bulk islet ATAC-seq across individual samples. (F) Fold enrichment (log_2_) of different sets of reproducible peaks from bulk islet ATAC-seq across 13 islet chromatin states. Genic enhancer is not shown because of no enrichment. (G) Overlap of cell-type peaks (alpha, beta, and predicted delta) with different sets of reproducible peaks from bulk islet ATAC-seq data.

To solve the challenge of learning cell-type-specific features from the sparse signal in the low-count delta cell cluster, we developed a novel a deep learning approach based on the U-Net architecture (Fig. S2A). U-Net was first developed for biomedical image segmentation but has since been applied to many other problems including audio and super resolution images. Its use in the super resolution problem served as the main impetus for our choice of model to upscale genomic signals. We formulated our approach as a classification problem in which we used sparse signal and corresponding peak calls to predict dense and high-quality peak calls. To avoid overfitting and create a robust, generalizable model, we adopted a rigorous training scheme. We divided the chromosomes into training, validation, and testing sets (Figure 3A, Figure S2B) and tested the performance of the models within the same cell type as well as across different cell types. We reasoned that our islet sci-ATAC-seq data were an ideal fit for this problem as all of the nuclei came from the same individual and processing batch and should, therefore, contain no genetic or technical biases that would influence within or across cell-type predictions. Because we had high-quality data from two cell types, we trained two models: one model was trained using 28-cell and 600-cell data from the alpha cells (alpha-trained model), while the second model was trained similarly on the data from the beta cells (beta-trained model). We then compared peak predictions from both models to corresponding MACS2 peaks from the 600-cell data. We found that the results from across cell-type predictions of both models outperformed the MACS2 peak results as measured by the mean average precision (Figure 3B), suggesting that the U-Net model was able to reconstruct peak calls from sparse signals independent of the specific cell type it was trained on. We highlight several examples in which the model was able to successfully predict peaks that were absent in the sparse 28-cell data but present in the 600-cell data of a cell type (Figure 3C). Because the training cell type had no signal or peak at the given locus, these predictions could not have been transferred or “copied over” from the training data, indicating a possible use across cell types or tissues. Based on these results, we used the U-Net models to predict peaks for the low-count delta cell cluster. As the U-Net model provides a posterior probability score for each peak call prediction, we sought to create a high-confidence set of predicted peak calls for each cell type. We used a threshold of 0.625 to filter the predicted peaks for each cell type. The choice of threshold was used to control for potential false positives and the final number of predicted cell-type peaks (Figs. S2C–D). Further, considering that the delta peak predictions from both the alpha and beta models were highly concordant (Jaccard index of 0.85), we used the intersection of the results as the final predicted outcome. We then validated our peak predictions using an orthogonal strategy in which we computed the enrichment of scRNA-seq derived signature genes (scRSGs) for the alpha, beta, and delta cells across chromatin accessibility peaks. We found that the scRSGs for each cell type consistently had higher enrichment in the predicted peaks than the MACS2 peaks derived from the same 28-cell data (Figure 3D), indicating that our predicted peaks captured cell-type specificity. In the next step, we compared them to the bulk islet ATAC-seq master peak calls (Table S5). We found that master peaks derived from the bulk islets were highly reproducible across samples, with >70% of the peaks occurring in five or more of the 10 samples (Figure 3E). Predictably, we also observed that the chromatin states corresponding to “active TSS” and “active enhancer” showed enrichment with increasing reproducibility of the master peaks. Likewise, chromatin states such as “repressed polycomb,” “weak transcription,” and “quiescent/low signal” showed a depletion with the increasing islet ATAC-seq peak reproducibility (Figure 3F). Similarly, when we compared the cell-type peaks to the master peaks, we found that the proportion of peaks from each cell type increased with the increasing reproducibility of the bulk peaks (Figure 3G), suggesting that highly reproducible peaks were driven by all of the constituent cell types while the peaks that occurred in fewer samples might have originated from underlying cell-population variability. For further validation, we also compared our cell-type peaks and sorted cell-type population peaks [19,20] with master peaks derived from 33 independent bulk islet ATAC-seq samples (Table S8) and observed a high degree of concordance (Fig. S2E). For example, >90% of the alpha and beta peaks were reproducible across three or more ATAC-seq samples, which was comparable to the 85–92% peak overlap observed for alpha and beta cell-type peaks from sorted cell populations in previous studies [19,20]. While the primary model of our interest was trained using data from 28 cells to predict 600-cell equivalent peaks, we asked if the model would perform similarly for a varying resolution of input data. To accomplish this, we subsampled cells from alpha and beta cell clusters to sets of different cell counts, starting with as few as five cells to 500 cells. We found that the performance of the model increased with the increasing number of cells used in the input training data (Fig. S2F). There was up to a five-fold gain in the coverage of the T2D GWAS SNPs in the beta predicted peaks compared to the MACS2 peaks (Fig. S2G) even when fewer cells were used as input training data (Fig. S2H). These results suggest that the deep learning strategy is applicable to a range of input data typically seen in single-cell sequencing experiments.

Overall, our results show that deep learning driven feature prediction can help recover tissue and cell-type relevant chromatin accessibility patterns from sparse and noisy data. Using this approach can enhance biological discoveries, which is challenging with rare cell populations.

### 3.4 T2D GWAS enrichment at cell-type-specific chromatin signatures

**Figure 4:**
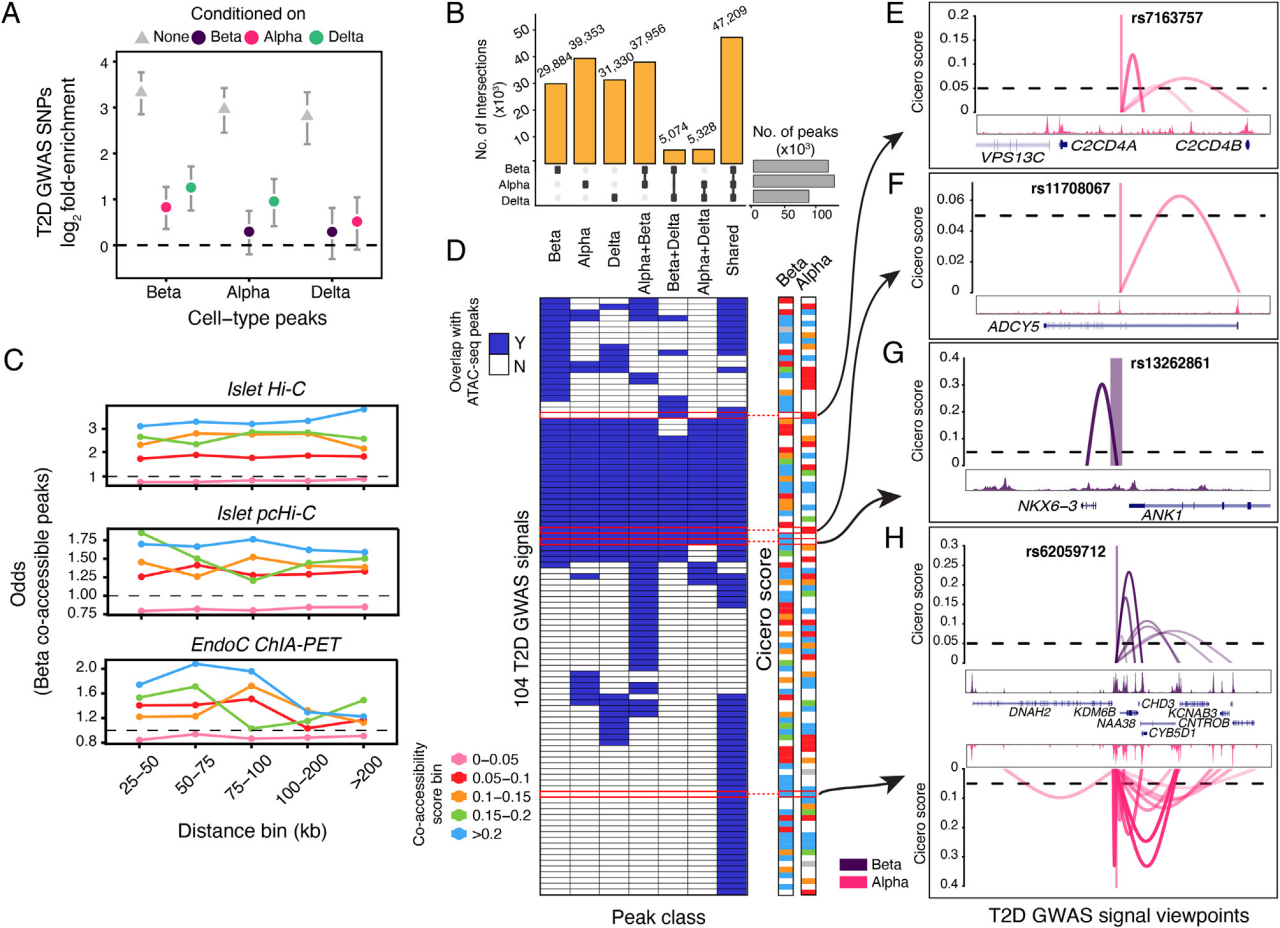
**Enrichment of T2D GWAS signals in cell-type-specific chromatin and linking them to target genes.** (A) Fold enrichment (log_2_) of T2D GWAS SNPs in cell-type peaks in single and conditional analysis mode using the fGWAS tool. For each cell type, three enrichment values with 95% confidence intervals are shown: none (single-annotation mode), alpha (conditioned on alpha), beta (conditioned on beta), and delta (conditioned on delta). (B) Partitioning of alpha, beta, and predicted delta peaks in mutually exclusive sets of cell-type-specific peaks. The subplot (on right) shows the total number of peaks for each cell type. (C) Distance-matched Fisher odds that beta cell co-accessibility links overlap islet Hi-C, islet pcHi-C, and ChIA-PET chromatin loops across different co-accessibility threshold bins. (D) Overlap of T2D GWAS credible set SNPs with cell-type-specific peaks. Bin is colored if there is at least one SNP (PPAg > 0.05) in the 99% genetic credible set of the T2D GWAS signal located within 1 kb of an ATAC-seq peak. The Cicero score columns are colored to indicate the score of the highest scoring link to the target gene. (E) Viewpoint plot of alpha Cicero connections centered at rs7163757 for *C2CD4A/B* locus. (F) Alpha Cicero connections centered at rs11708067 for *ADCY5* locus. (G) Beta Cicero connections centered at rs13262861 for *ANK1* locus. (H) Cicero connections for both alpha and beta centered at rs62059712 for *ATP1B2* locus. The viewpoint region spans ±1 kb from the variant.

We computed the overlap enrichment of T2D GWAS loci in cell-type peak annotations from the alpha, beta, and delta cells using a Bayesian hierarchical model as implemented in fGWAS [46]. fGWAS allows calculation of marginal enrichment associations for one cell type conditioned on another using not only the subset of genome-wide significant loci but also the full genome-wide association summary statistics. We observed that annotations from all three cell types were highly enriched for T2D GWAS loci, with beta-cell annotations having the highest enrichment values (Figure 4A). However, when we accounted for marginal associations using a joint model, we found that the beta cells were the only cell type to remain enriched after adjusting for the other two cell types. This result suggests that shared or beta cell-specific chromatin accessibility peaks drive the association with T2D GWAS. More broadly, these findings illustrate how single-cell chromatin profiling results, when coupled with conditional statistical enrichment analyses, can dissect specific cell types that drive enrichment in bulk tissue samples.

We next partitioned the peaks into exclusive sets based on the cell types shared by each peak. Because the delta cell cluster had fewer reads compared to the alpha and beta cells, we did not utilize read-count-based approaches to determine cell-type-specific peaks. Instead, we used peak level metrics to identify peaks exclusive to a combination of cell types. We found that a majority of the peaks (47,209) were shared across all cell types and that each cell type had a set of unique accessible sites (29,884 beta, 39,353 alpha, and 31,330 delta) (Figure 4B). Consistent with our expectations, TSS-proximal shared peaks mostly overlapped active TSS chromatin states compared to cell-type-specific peaks that had a larger proportion of peaks in active enhancer states (Fig. S3A). To further understand the regulatory logic, we looked for TF motifs enriched in cell-type-specific peaks using GAT [30]. We found enrichment of motifs implicated in islet cell-type-specific functionality consistent with known islet TFs (Fig. S3B). For example, PDX1 was enriched in beta-specific peaks, while GATA6 and FOXA were enriched in alpha-specific peaks. We also observed enrichment of motifs relevant to endocrine function such as PAX6 and MAF. For delta cell peaks, HHEX was the only TF signature gene (out of 17 scRNA-seq cell-type signature genes) that encoded a transcription factor, but we observed no delta-specific enrichment. We think this could be because HHEX is a member of the homeobox family of TFs and therefore has a highly degenerate motif, which could result in less specific enrichment within delta-specific peaks. Overall, the alpha and beta peak motif enrichments were consistent with known cell-specific TFs. We then used a complementary enrichment approach with the GREGOR tool [45] to determine if T2D GWAS loci were enriched in each subclass of peaks. We found that the T2D GWAS loci were highly enriched in shared peaks (p-value = 1.64e-16, fold enrichment = 2.03) and beta cell-specific peaks (p-value = 6.42e-6, fold enrichment = 1.91) (Fig. S3C). We also observed moderate enrichment of T2D GWAS SNPs in other sets of cell-type-specific peaks, but strikingly, there was little enrichment in delta cell-specific peaks (p-value = 3.12e-3, fold enrichment = 1.55) and no significant enrichment in alpha cell-specific peaks (p-value = 1.83e-01, fold enrichment = 1.16). This suggests that the role of alpha and delta cells in the mechanisms underlying genetic predisposition to T2D pathophysiology might be limited compared to beta cells. To further elucidate the role of shared peaks in islet endocrine-specific peaks and constitutive peaks shared across more broad cell types, we added peaks from a sorted acinar cell population. We observed that acinar-specific peaks showed no enrichment (p-value = 0.31, fold enrichment = 1.10) (Fig. S3D). These independent enrichment findings from the GREGOR tool were consistent with the results of the fGWAS analysis (Figure 4A), indicating the robust nature of these results.

### 3.5 Linking cell-type-specific chromatin accessibility to target genes

One of the primary challenges in understanding the underlying biological mechanisms of non-coding T2D GWAS variants is the identification of their target genes. Risk variants occurring in enhancer regions can often interact with their target genes that are not adjacent. Multiple studies have examined the regulatory landscape of pancreatic islets and relevant cell lines using chromosome conformation capture techniques to nominate target genes [[51], [52], [53]]. However, most of these studies were conducted on bulk islet samples, thereby obscuring any cell-specific signatures of chromatin looping. Additionally, chromatin looping studies tend to have noisy signals when two regions are close in linear space, which leads to a bias toward detecting longer–range interactions. To mitigate these limitations, we adopted a recently published approach, Cicero [50], which leverages profiles of chromatin co-accessibility across single cells to infer pairs of chromatin peaks that are likely to be in close physical proximity. For this analysis, we focused on alpha and beta cell types as they were the clusters with the most nuclei. To filter the Cicero co-accessibility scores for the peak pairs that are more likely to represent true looping, we compared our results to experimentally defined loops from three orthogonal and independent chromatin looping datasets: islet Hi-C [51], islet promoter capture Hi-C (pcHi-C) [52], and EndoC Pol2 ChIA-PET [53] loops. We found that Cicero peak pairs from our sci-ATAC-seq data with scores >0.05 were strongly enriched to be considered loops in each of the three reference data sets (Figure 4C and Figure S4A). Using this threshold, we found 190,176 beta cell and 147,716 alpha cell co-accessible peak pairs.

Using our new catalog of Cicero-inferred chromatin loops, we next sought to link TSS-distal T2D GWAS variants to target gene promoters. We focused on the latest T2D GWAS results and used SNPs in association signals that were genetically fine-mapped to be in a 99% credible set and had a >0.05 posterior probability of association (PPAg) [3]. For this mapping procedure, we required that the credible set SNP was not within 1 kb of an annotated TSS and that the other end of the chromatin loop occurred within 1 kb of an annotated TSS. Using this approach across both alpha and beta cells, we found that of the 265 independent GWAS signals containing SNPs that met our criteria, we were able to nominate target genes at 104 of them (Figure 4D). In a similar manner, we checked if the SNPs within each locus overlapped a cell-type-specific peak (Table S6). We observed several notable examples. At the *C2CD4A/B* locus, we found rs7163757 (PPAg 0.095) to be linked to *C2CD4B* in the alpha cells (Figure 4E). Using an islet gene expression and genetic integration approach to identify expression quantitative trait loci (eQTL), we previously showed that rs7163757 is associated with *C2CD4B* expression [8], and a subsequent functional study corroborated these findings [58]. At a different locus, we found rs11708067 (PPAg 0.79) located in an islet enhancer within the *ADCY5* gene to be linked to the TSS of the corresponding gene (Figure 4F). The risk allele of rs11708067 has been reported to be associated with reduced expression of *ADCY5* [59], and functional validation experiments show an association with impaired insulin secretion [4,9]. As an example of a beta cell-specific connection, we found variant rs13262861 (PPAg 0.97) within the *ANK1* locus to be linked to nearby *NKX6-3* (Figure 4G). We previously used islet eQTL data to nominate *NKX6-3* as an islet target gene at this locus [4,8]. The extensive support from previous publications for these three loci serves as positive controls for our results and reinforces the quality of this sci-ATAC-seq data and analyses. Finally, we highlight rs62059712 (PPAg 0.34) within the *ATP1B2* locus as an example of a variant linked to multiple gene promoters across both beta and alpha cell types (Figure 4H). Notably, of the 104 T2D GWAS signals for which we were able to nominate target genes in either cell type, 60 (∼58%) had more than one nominated target gene.

## 4 Discussion

Single-nuclei chromatin accessibility profiling provides a unique approach for mapping cell-type-specific regulatory signatures. In this study, we utilized the sci-ATAC-seq protocol to generate and study chromatin accessibility profiles for 1,456 high-quality nuclei from a purified pancreatic islet sample. Our dataset and analyses provide high-quality maps of cell-type accessibility profiles and regulatory architecture using an unbiased approach compared to prior maps from sorted cell-type populations. Our validation analyses span data from seven independent publications and encompass different aspects of our experiments and computational analyses and uniformly support the reproducibility of our data and results. However, it is essential to emphasize that single-cell data present unique challenges, and that our study, which analyzed only one pancreatic islet sample, may be limited in how it can address some problems.

First, de novo identification of cell types and within-cluster heterogeneity from sparse single-cell chromatin accessibility data continues to be a challenge. We adopted several strategies to address potential biases in our analyses. Our logistic regression approach to eliminate read depth as a confounding technical variable, combined with the binomial counting strategy to infer doublet enrichment in clusters, enabled us to identify three major cell-type populations corresponding to alpha, beta, and delta cells. To assign these cell identities, we relied not only on classical hormone markers but also leveraged findings from independent islet single-cell RNA-seq studies to validate our results. While islets have been reported to contain other rarer cell-type populations (<5% of all islet cells) [56], our ability to observe them was limited due to the size of our dataset. We were also limited in our ability to analyze within-cluster heterogeneity due to relatively low number of cells in each cluster (for instance, only 701 beta cells) and the availability of only one sample.

Second, we faced the challenge of identifying reliable cell-specific accessibility patterns across all cell types due to the relatively low abundance of delta cells. As such, our U-Net-based deep learning approach presents a novel strategy for addressing this particular problem. Our model differs from a related deep learning method, Coda [60], by focusing on single-cell ATAC-seq as opposed to bulk histone ChIP-seq data and uses a more complex architecture (U-Net) that has been previously used in image processing related tasks [40,61] but has been seldom used in genomics [62]. Using alpha and beta cells as reciprocal training and testing datasets, we demonstrated that our model successfully learns to predict high-quality peak calls from low cell count data. We validate our upscaled peaks using orthogonal data from independent scRNA-seq study and show that upscaled peaks have higher enrichment of cell-type signature genes compared to MACS2 peaks. However, there are diminishing returns from using deep learning models when 200 or more cells are used as input to the model, an observation consistent with the threshold of experimental reproducibility highlighted in a recent large-scale single-nuclei ATAC-seq study [15]. This consistency with an independent study reinforces the value of our deep learning approach but also highlights a limitation of our delta peak predictions that derive from a low cell count input dataset. Our observation also provides a strategy for experimental design in which a certain number of samples might be desirable to ensure a minimum performance. We envision that our method will be useful under scenarios in which it is challenging or cost-prohibitive to obtain specific cell populations.

Overall, an important implication of our findings comes from our ability to generate cell-specific chromatin accessibility maps and infer looping connections from accessible regions to target genes of T2D GWAS variants. A recent T2D GWAS [3] reported > 400 independent association signals, but the molecular mechanisms underlying these signals was known only for a subset of the variants. Single-nuclei resolution cell-specific regulatory signatures provide a unique opportunity to infer target gene links with non-coding elements. Thus, we integrated cell-type co-accessibility links with T2D GWAS SNPs that were genetically fine-mapped to 99% credible sets to create a higher-resolution map of the regulatory landscape underlying 104 distinct T2D GWAS signals. Focusing on the cell specificity of the chromatin accessibility peaks that anchor these target gene associations, we observed seven classes, representing: i) peaks that were unique to a cell type (three classes), ii) peaks that were shared across all three cell types (one class), and iii) peaks that occurred in a pair of cell types (three classes). Interestingly, the class of peaks shared across all three cell types comprised 26 of the 104 (25%) T2D GWAS to target gene links even though this class was only one of seven. These results paint a complex picture of disease mechanisms in which certain risk variants may mediate target effects through cell-type-specific pathways, while others might affect multiple target genes shared across cell-type populations.

We noted specific examples at the *C2CD4A/B* and *ANK1* loci in which we were able to nominate specific variants linked with islet gene expression and their role in T2D pathophysiology as compelling targets for future mechanistic studies. At the time of writing this manuscript, another similar study appeared as a preprint [63], and as such, an important future topic will be to combine and meta-analyze multiple islet single-cell ATAC-seq datasets. Such an endeavor will increase statistical power to detect chromatin features, including loops, at GWAS loci, and eventually enable single-cell resolution chromatin QTL studies, which will help to further focus on functional SNPs. Overall, we believe that the data, results, and methodology from this study will be of value to the broader research community.

## 5 Data availability

The data reported in this paper were deposited in the database of Genotypes and Phenotypes (accession no. phs001188.v2.p1; FUSION Tissue Biopsy Study-Islet Expression and Regulation by RNAseq and ATACseq). The code for deep learning analysis is available on GitHub at https://github.com/ParkerLab/PillowNet. Custom scripts are available at https://github.com/ParkerLab/islet_sci-ATAC-seq_2019. Additional URLs are provided in Table S7 of the Supplementary Data.

## Author contributions

FSC, JS, and SCJP conceived the study. MRE, DC, and RMD generated the data. LSZ, JPD, and NN performed the initial analysis. VR, DXQ, and SCJP analyzed the data and conducted the research. DXQ and YG implemented the U-Net strategy and wrote the code. FSC, JS, and SCJP jointly supervised the work. VR, DXQ, and SCJP wrote the manuscript with feedback from all of the authors.

## References

[1] DeFronzo R.A., Ferrannini E., Groop L., Henry R.R., Herman W.H., Holst J.J. (2015). Type 2 diabetes mellitus. Nature reviews Disease primers.

[2] Spellman C.W. (2010). Pathophysiology of type 2 diabetes: targeting islet cell dysfunction. Journal of the American Osteopathic Association.

[3] Mahajan A., Taliun D., Thurner M., Robertson N.R., Torres J.M., Rayner N.W. (2018). Fine-mapping type 2 diabetes loci to single-variant resolution using high-density imputation and islet-specific epigenome maps. Nature Genetics.

[4] Viñuela A., Varshney A., van de Bunt M., Prasad R.B., Asplund O.B., Bennett A. (2019). Influence of genetic variants on gene expression in human pancreatic islets – implications for type 2 diabetes. BioRxiv.

[5] Parker S.C.J., Stitzel M.L., Taylor D.L., Orozco J.M., Erdos M.R., Akiyama J.A. (2013). Chromatin stretch enhancer states drive cell-specific gene regulation and harbor human disease risk variants. Proceedings of the National Academy of Sciences.

[6] Pasquali L., Gaulton K.J., Rodríguez-Seguí S.A., Mularoni L., Miguel-Escalada I., Akerman İ. (2014). Pancreatic islet enhancer clusters enriched in type 2 diabetes risk-associated variants. Nature Genetics.

[7] Thurner M., van de Bunt M., Torres J.M., Mahajan A., Nylander V., Bennett A.J. (2018). Integration of human pancreatic islet genomic data refines regulatory mechanisms at Type 2 Diabetes susceptibility loci. ELife.

[8] Varshney A., Scott L.J., Welch R.P., Erdos M.R., Chines P.S., Narisu N. (2017). Genetic regulatory signatures underlying islet gene expression and type 2 diabetes. Proceedings of the National Academy of Sciences.

[9] van de Bunt M., Fox J.E.M., Dai X., Barrett A., Grey C., Li L. (2015). Transcript expression data from human islets links regulatory signals from genome-wide association studies for type 2 diabetes and glycemic traits to their downstream effectors. PLoS Genetics.

[10] Buenrostro J.D., Giresi P.G., Zaba L.C., Chang H.Y., Greenleaf W.J. (2013). Transposition of native chromatin for fast and sensitive epigenomic profiling of open chromatin, DNA-binding proteins and nucleosome position. Nature Methods.

[11] Hesselberth J.R., Chen X., Zhang Z., Sabo P.J., Sandstrom R., Reynolds A.P. (2009). Global mapping of protein-DNA interactions in vivo by digital genomic footprinting. Nature Methods.

[12] Shema E., Bernstein B.E., Buenrostro J.D. (2018). Single-cell and single-molecule epigenomics to uncover genome regulation at unprecedented resolution. Nature Genetics.

[13] Cusanovich D.A., Reddington J.P., Garfield D.A., Daza R.M., Aghamirzaie D., Marco-Ferreres R. (2018). The cis-regulatory dynamics of embryonic development at single-cell resolution. Nature.

[14] Preissl S., Fang R., Huang H., Zhao Y., Raviram R., Gorkin D.U. (2018). Single-nucleus analysis of accessible chromatin in developing mouse forebrain reveals cell-type-specific transcriptional regulation. Nature Neuroscience.

[15] Satpathy A.T., Granja J.M., Yost K.E., Qi Y., Meschi F., McDermott G.P. (2019). Massively parallel single-cell chromatin landscapes of human immune cell development and intratumoral T cell exhaustion. Nature Biotechnology.

[16] Cusanovich D.A., Hill A.J., Aghamirzaie D., Daza R.M., Pliner H.A., Berletch J.B. (2018). A single-cell Atlas of in vivo mammalian chromatin accessibility. Cell.

[17] Lawlor N., George J., Bolisetty M., Kursawe R., Sun L., Sivakamasundari V. (2017). Single-cell transcriptomes identify human islet cell signatures and reveal cell-type–specific expression changes in type 2 diabetes. Genome Research.

[18] Muraro M.J., Dharmadhikari G., Grün D., Groen N., Dielen T., Jansen E. (2016). A single-cell transcriptome Atlas of the human pancreas. Cells Systems.

[19] Arda H.E., Tsai J., Rosli Y.R., Giresi P., Bottino R., Greenleaf W.J. (2018). A chromatin basis for cell lineage and disease risk in the human pancreas. Cells Systems.

[20] Ackermann A.M., Wang Z., Schug J., Naji A., Kaestner K.H. (2016). Integration of ATAC-seq and RNA-seq identifies human alpha cell and beta cell signature genes. Molecular Metabolism.

[21] Nica A.C., Ongen H., Irminger J.-C., Bosco D., Berney T., Antonarakis S.E. (2013). Cell-type, allelic, and genetic signatures in the human pancreatic beta cell transcriptome. Genome Research.

[22] Dorrell C., Grompe M.T., Pan F.C., Zhong Y., Canaday P.S., Shultz L.D. (2011). Isolation of mouse pancreatic alpha, beta, duct and acinar populations with cell surface markers. Molecular and Cellular Endocrinology.

[23] Cusanovich D.A., Daza R., Adey A., Pliner H.A., Christiansen L., Gunderson K.L. (2015). Multiplex single-cell profiling of chromatin accessibility by combinatorial cellular indexing. Science.

[24] Hensley John (2017). cta: C++ implementation of Buenrostro adapter trimming.

[25] Li H. (2013). Aligning sequence reads, clone sequences and assembly contigs with BWA-MEM.

[26] Li H., Handsaker B., Wysoker A., Fennell T., Ruan J., Homer N. (2009). The sequence alignment/map format and Samtools. Bioinformatics.

[27] Zhang Y., Liu T., Meyer C.A., Eeckhoute J., Johnson D.S., Bernstein B.E. (2008). Model-based analysis of ChIP-seq (MACS). Genome Biology.

[28] The ENCODE Project Consortium (2012). An integrated encyclopedia of DNA elements in the human genome. Nature.

[29] (2018). A toolkit for QC and visualization of ATAC-seq results.: ParkerLab/ataqv.

[30] Heger A., Webber C., Goodson M., Ponting C.P., Lunter G. (2013). GAT: a simulation framework for testing the association of genomic intervals. Bioinformatics.

[31] Bolger A.M., Lohse M., Usadel B. (2014). Trimmomatic: a flexible trimmer for Illumina sequence data. Bioinformatics.

[32] Fraley C., Raftery A.E., Scrucca L., Murphy T.B., Fop M. (2019). Mclust: Gaussian mixture modelling for model-based clustering, classification, and density estimation.

[33] O'Leary N.A., Wright M.W., Brister J.R., Ciufo S., Haddad D., McVeigh R. (2016). Reference sequence (RefSeq) database at NCBI: current status, taxonomic expansion, and functional annotation. Nucleic Acids Research.

[34] Liao Y., Smyth G.K., Shi W. (2013). The Subread aligner: fast, accurate and scalable read mapping by seed-and-vote. Nucleic Acids Research.

[35] Gierahn T.M., Wadsworth Ii M.H., Hughes T.K., Bryson B.D., Butler A., Satija R. (2017). Seq-Well: portable, low-cost RNA sequencing of single cells at high throughput. Nature Methods.

[36] UMAP (2018). Uniform manifold approximation and projection. J Open Source Software.

[37] Hahsler M., Piekenbrock M., Arya S., Mount D. (2019). Dbscan: density based clustering of applications with noise (DBSCAN) and related algorithms.

[38] Harrow J., Frankish A., Gonzalez J.M., Tapanari E., Diekhans M., Kokocinski F. (2012). GENCODE: the reference human genome annotation for the ENCODE Project. Genome Research.

[39] Segerstolpe Å., Palasantza A., Eliasson P., Andersson E.-M., Andréasson A.-C., Sun X. (2016). Single-cell transcriptome profiling of human pancreatic islets in Health and type 2 diabetes. Cell Metabolism.

[40] Ronneberger O., Fischer P., Brox T. (2015). U-Net: convolutional networks for biomedical image segmentation.

[41] Chollet François (2015). Keras. GitHub.

[42] Abadi M., Barham P., Chen J., Chen Z., Davis A., Dean J. (2016). TensorFlow: a system for large-scale machine learning.

[43] Kingma D.P., Ba J. (2014). Adam: a method for stochastic optimization.

[44] Quinlan A.R., Hall I.M. (2010). BEDTools: a flexible suite of utilities for comparing genomic features. Bioinformatics.

[45] Schmidt E.M., Zhang J., Zhou W., Chen J., Mohlke K.L., Chen Y.E. (2015). GREGOR: evaluating global enrichment of trait-associated variants in epigenomic features using a systematic, data-driven approach. Bioinformatics.

[46] Pickrell J.K. (2014). Joint analysis of functional genomic data and genome-wide association studies of 18 human traits. The American Journal of Human Genetics.

[47] Scott L.J., Erdos M.R., Huyghe J.R., Welch R.P., Beck A.T., Wolford B.N. (2016). The genetic regulatory signature of type 2 diabetes in human skeletal muscle. Nature Communications.

[48] The 1000 Genomes Project Consortium (2015). A global reference for human genetic variation. Nature.

[49] Grant C.E., Bailey T.L., Noble W.S. (2011). FIMO: scanning for occurrences of a given motif. Bioinformatics.

[50] Pliner H.A., Packer J.S., McFaline-Figueroa J.L., Cusanovich D.A., Daza R.M., Aghamirzaie D. (2018). Cicero predicts cis-regulatory DNA interactions from single-cell chromatin accessibility data. Molecular Cell.

[51] Greenwald W.W., Li H., Benaglio P., Jakubosky D., Matsui H., Schmitt A. (2019). Subtle changes in chromatin loop contact propensity are associated with differential gene regulation and expression. Nature Communications.

[52] Miguel-Escalada I., Bonàs-Guarch S., Cebola I., Ponsa-Cobas J., Mendieta-Esteban J., Atla G. (2019). Human pancreatic islet three-dimensional chromatin architecture provides insights into the genetics of type 2 diabetes. Nature Genetics.

[53] Lawlor N., Márquez E.J., Orchard P., Narisu N., Shamim M.S., Thibodeau A. (2019). Multiomic profiling identifies cis-regulatory networks underlying human pancreatic β cell identity and function. Cell Reports.

[54] Hnisz D., Abraham B.J., Lee T.I., Lau A., Saint-André V., Sigova A.A. (2013). Super-enhancers in the control of cell identity and disease. Cell.

[55] Bulger M., Groudine M. (2011). Functional and mechanistic diversity of distal transcription enhancers. Cell.

[56] Cabrera O., Berman D.M., Kenyon N.S., Ricordi C., Berggren P.-O., Caicedo A. (2006). The unique cytoarchitecture of human pancreatic islets has implications for islet cell function. Proceedings of the National Academy of Sciences.

[57] Brissova M., Fowler M.J., Nicholson W.E., Chu A., Hirshberg B., Harlan D.M. (2005). Assessment of human pancreatic islet architecture and composition by laser scanning confocal microscopy. Journal of Histochemistry and Cytochemistry.

[58] Kycia I., Wolford B.N., Huyghe J.R., Fuchsberger C., Vadlamudi S., Kursawe R. (2018). A common type 2 diabetes risk variant potentiates activity of an evolutionarily conserved islet stretch enhancer and increases C2CD4A and C2CD4B expression. The American Journal of Human Genetics.

[59] Roman T.S., Cannon M.E., Vadlamudi S., Buchkovich M.L., Wolford B.N., Welch R.P. (2017). A type 2 diabetes-associated functional regulatory variant in a pancreatic islet enhancer at the ADCY5 locus. Diabetes.

[60] Koh P.W., Pierson E., Kundaje A. (2017). Denoising genome-wide histone ChIP-seq with convolutional neural networks. Bioinformatics.

[61] Falk T., Mai D., Bensch R., Çiçek Ö., Abdulkadir A., Marrakchi Y. (2019). U-Net: deep learning for cell counting, detection, and morphometry. Nature Methods.

[62] Eraslan G., Avsec Ž., Gagneur J., Theis F.J. (2019). Deep learning: new computational modelling techniques for genomics. Nature Reviews Genetics.

[63] Chiou J., Zeng C., Cheng Z., Han J.Y., Schlichting M., Huang S. (2019). Single cell chromatin accessibility reveals pancreatic islet cell type- and state-specific regulatory programs of diabetes risk. BioRxiv.

