# Supplementary material for: Single-cell ATAC-Seq in human pancreatic islets and deep learning upscaling of rare cells reveals cell-specific type 2 diabetes regulatory signatures
Source: Mol Metab. 2019 Dec 20;32:109–21. doi: 10.1016/j.molmet.2019.12.006 (PMC6961712; doi:10.1016/j.molmet.2019.12.006)
Supplement: Multimedia component 2 [file mmc2.pdf]

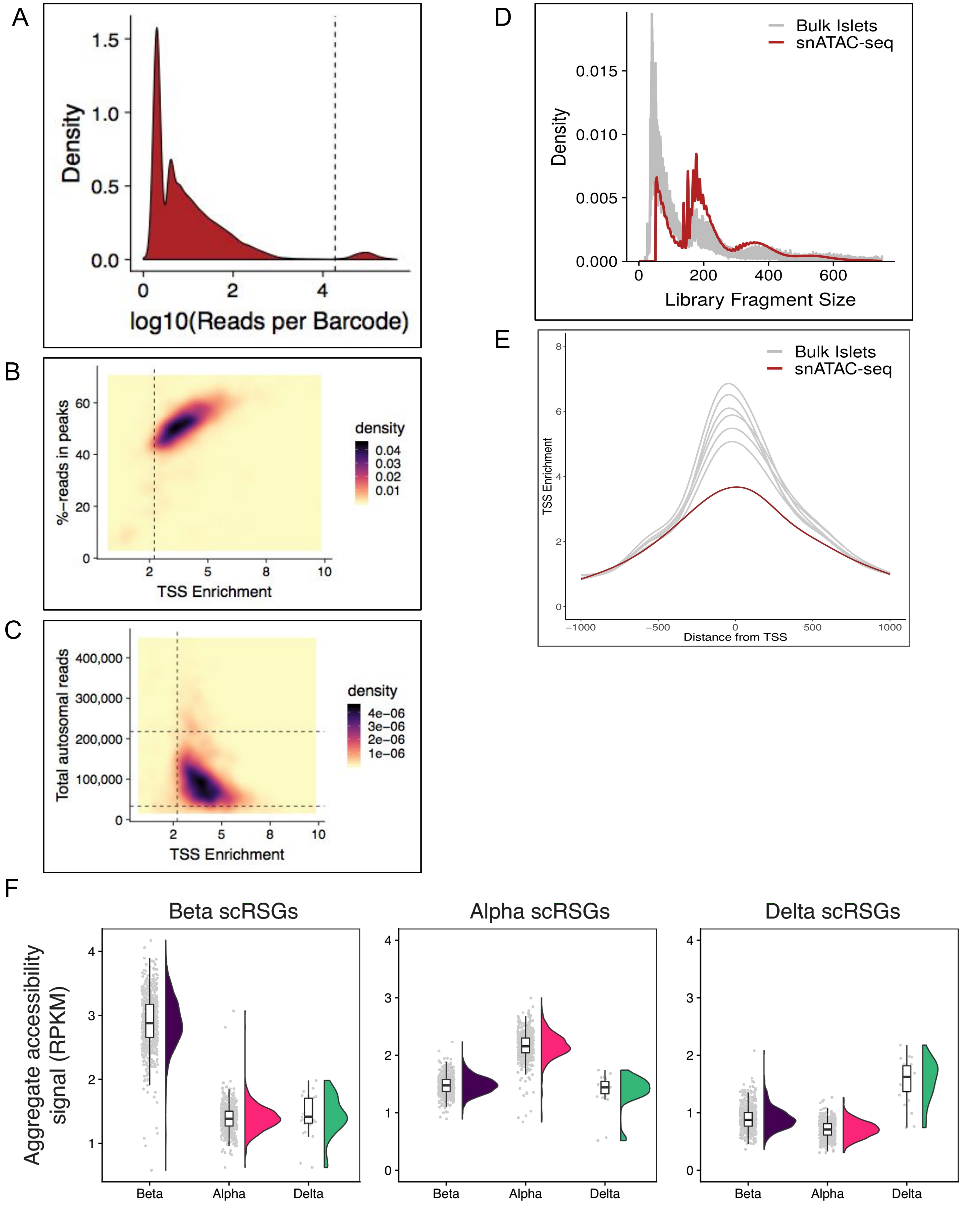

Figure S1. ATAC-seq metrics of nuclei from sci-ATAC-seq. (A) Distribution of reads per barcodes shown with the threshold chosen for filtering background barcodes. (B) Fraction of reads in peaks versus TSS Enrichment, and (C) Total autosomal reads versus TSS enrichment for all single-nuclei. Density units are arbitrary. (D) TSS coverage of aggregate sci-ATAC-seq, and (E) Fragment length distribution of aggregate sci-ATAC-seq compared with ten bulk islet ATAC-seq samples. (F) Chromatin accessibility signal in single-cell RNA-seq derived cell-type signature genes (scRSGs; Beta=83, Alpha=168, Delta=53) across three sci-ATAC-seq identified cell clusters. scRSGS obtained from Segerstolpe et al [39].

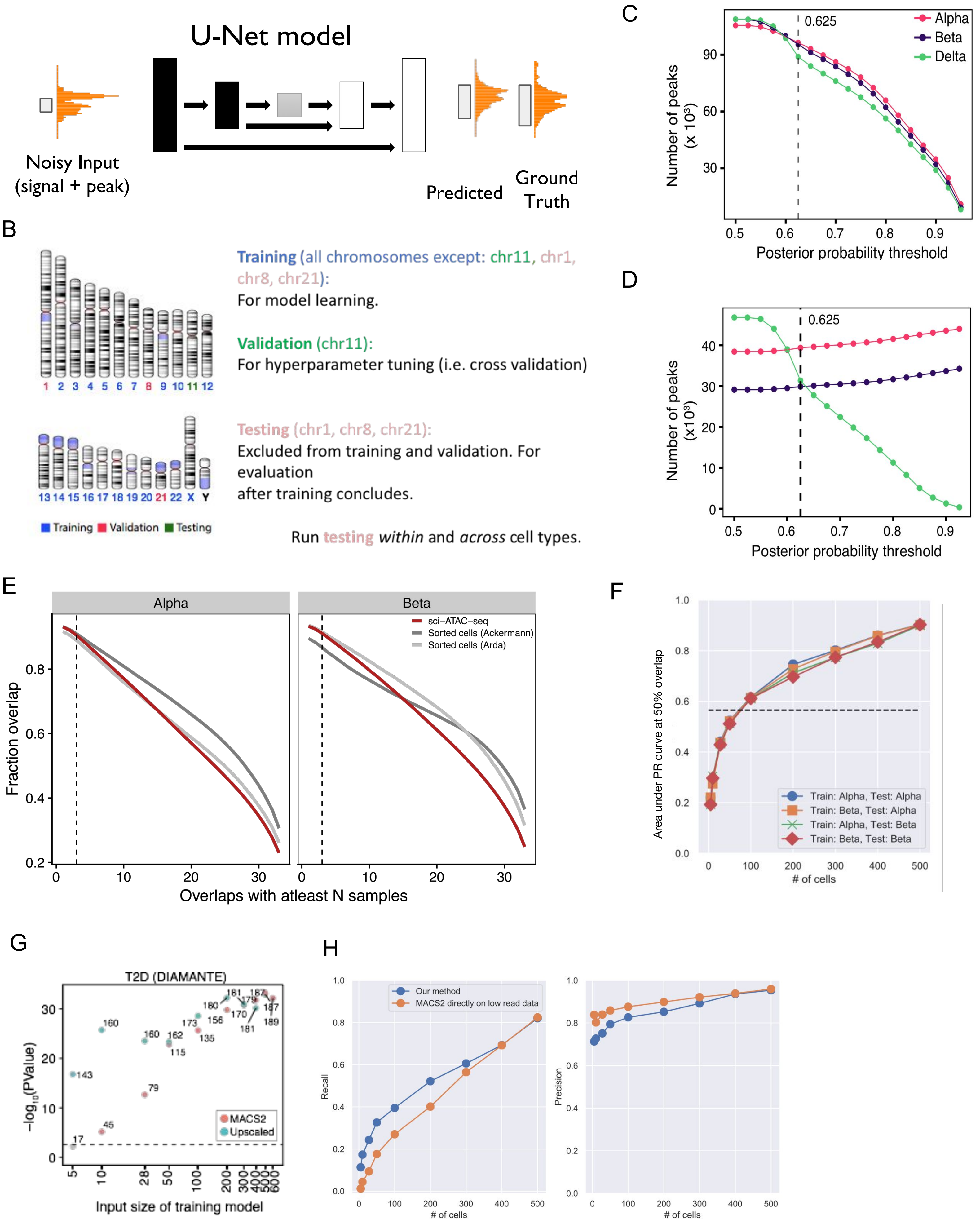
U-Net model
Noisy Input
(signal + peak)
Predicted
Ground
Truth
B
Training (all chromosomes except: chr11, chr1, chr8, chr21):
For model learning.
Validation (chr11):
For hyperparameter tuning (i.e. cross validation)
Testing (chr1, chr8, chr21):
Excluded from training and validation. For evaluation
after training concludes.
1 2 3 4 5 6 7 8 9 10 11 12
13 14 15 16 17 18 19 20 21 22 X Y
Training Validation Testing
Run testing within and across cell types.
C
0.625
Alpha
Beta
Delta
Number of peaks
(x 10³)
Posterior probability threshold
D
0.625
Number of peaks
(x10³)
Posterior probability threshold
E
Alpha
Beta
sci−ATAC−seq
Sorted cells (Ackermann)
Sorted cells (Arda)
Fraction overlap
Overlaps with atleast N samples
F
Area under PR curve at 50% overlap
Train: Alpha, Test: Alpha
Train: Beta, Test: Alpha
Train: Alpha, Test: Beta
Train: Beta, Test: Beta
# of cells
G
T2D (DIAMANTE)
−log₁₀(PValue)
MACS2
Upscaled
Input size of training model
H
Our method
MACS2 directly on low read data
Recall
Precision
# of cells

Figure S2. Peak calling using deep learning approach. (A) Schematic of U-Net learning strategy. (B) The training, testing, and validation scheme used for training the models delineating which chromosomes were part of what dataset. (C) Number of predicted peaks (from 28-cell trained model) for each cell type with different output posterior probability thresholds. (D) Number of cell-type specific peaks for alpha, beta, and delta after partitioning into mutually exclusive sets (see methods) with different output posterior probability thresholds. (E) Fraction overlap of cell-type peaks (alpha, beta) from our study and sorted cell populations from Ackermann et al (2016) and Arda et al (2018) with different sets of reproducible bulk islet ATAC-seq peaks obtained from 33 bulk islet ATAC-seq samples. (F) Average precision in predicting peaks compared for all four models (two training and two prediction datasets) with different sizes of input training data. (G) Enrichment of T2D GWAS SNPs (N=378) in predicted beta peak calls (from alpha-trained model) compared with peaks calls from MACS2 on the data with varying size of input training data. (H) Precision and recall curves comparing predicted beta peaks (from alpha-trained model) for varying size of input training data.

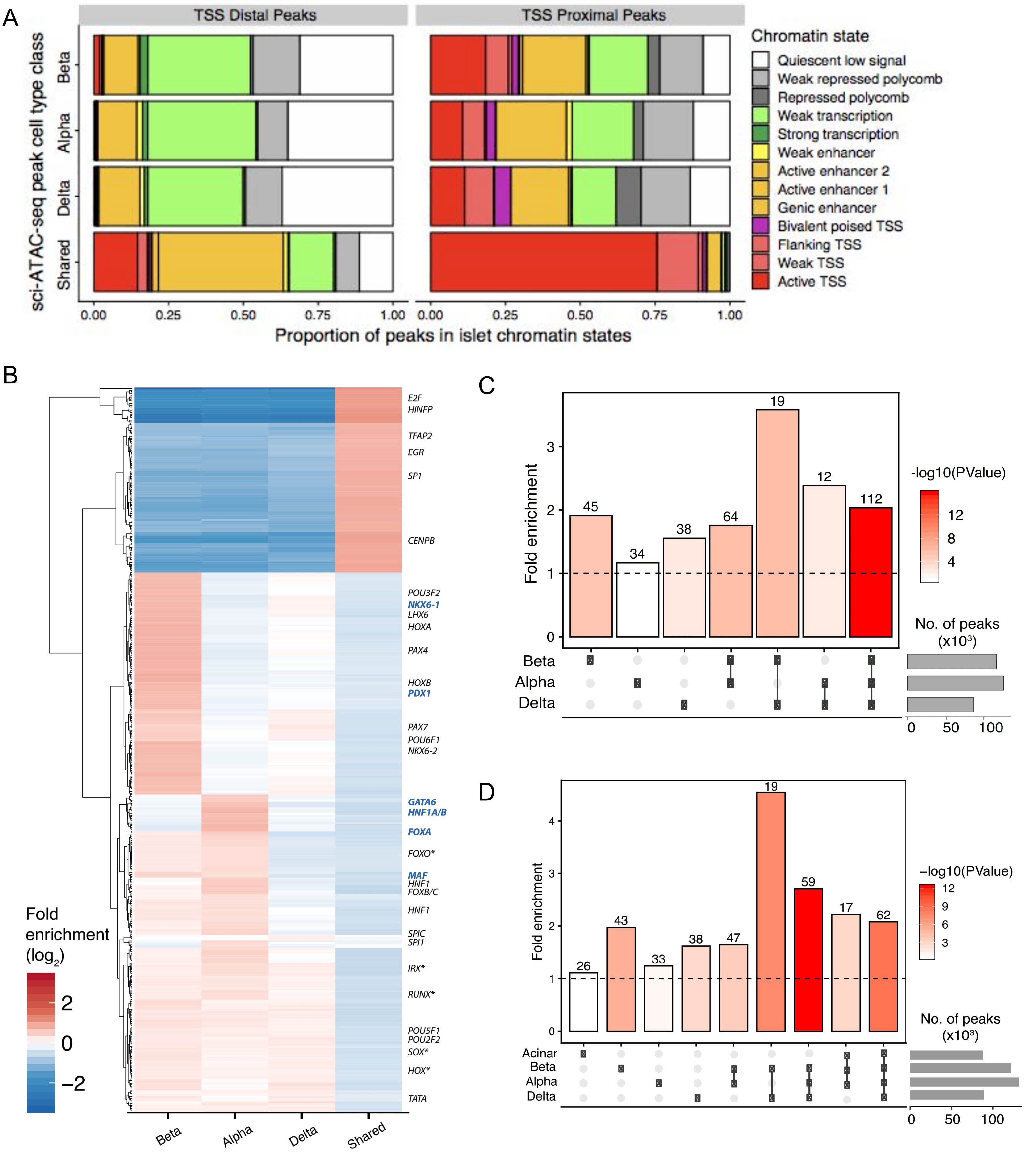

Figure S3. (A) Distribution of TSS proximal and distal peaks (>5kb from nearest Refseq TSS) in shared peaks and peaks assigned only to alpha, beta, and delta cell-type. (B) Transcription factor (TF) motif enrichment ($\log_2$) across cell-type specific and shared peaks. (C) Enrichment of T2D GWAS SNPs (N=378) across all cell-type specific sets of peaks. (D) Enrichment of T2D GWAS SNPs (N=378) across all cell-type specific sets of peaks including peaks from *acinar cells.*

A

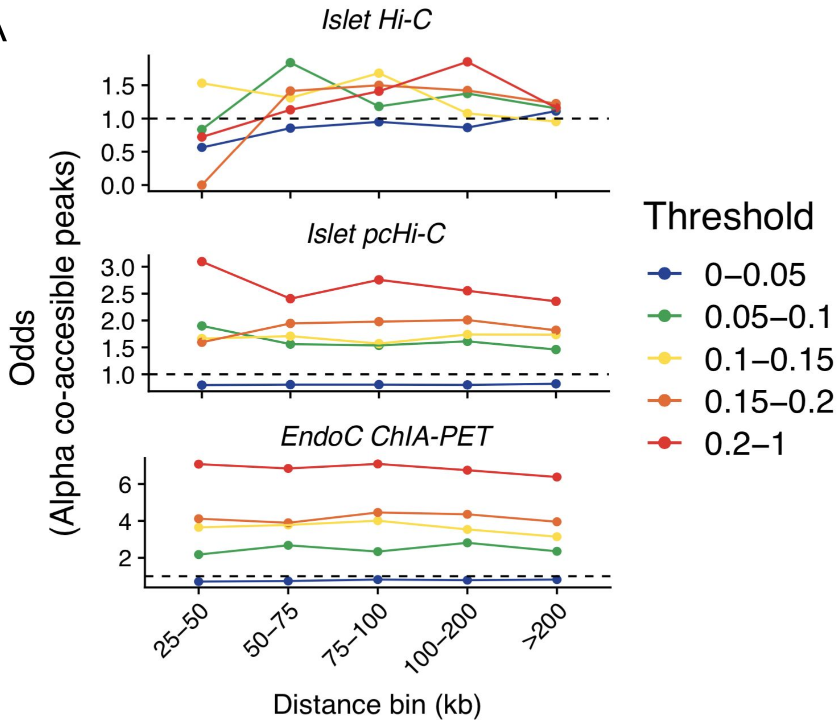

Figure S4. (A) Fisher odds score for enrichment of alpha co-accessible sites in loop anchors from three different dataset: Islet Hi-C, Islet pcHi-C, and EndoC ChIA-PET.
